# A Platelet Acquired Storage Pool Disorder Associated with Tamoxifen Therapy

**DOI:** 10.1155/2012/948351

**Published:** 2012-12-26

**Authors:** Lalitha Nayak, Alvin H. Schmaier

**Affiliations:** Seidman Cancer Center, University Hospitals Case Medical Center and Division of Hematology and Oncology, Department of Medicine, Case Western Reserve University, 10900 Euclid Avenue, WRB2-130, Cleveland, OH 44106-7284, USA

## Abstract

The antiestrogenic drug tamoxifen, used in patients with breast cancer, is associated with an increase in arterial and venous thrombotic events, the mechanism of which is not clearly understood. We report a case of a lady who presented with new bruising and prolonged bleeding following a tooth extraction 4–6 weeks after starting tamoxifen. Investigations were consistent with an acquired platelet storage pool disorder. Repeat platelet function analysis was normal, performed 3 months after discontinuation of tamoxifen. We present a previously clinically unreported effect of tamoxifen on platelet function.

## 1. Introduction

Tamoxifen, an antiestrogenic drug belonging to the selective estrogen receptor modulator (SERM), is widely used for the prevention and treatment of breast cancer. It induces apoptosis in breast cancer cells through caspase-3 and JNK-1 pathways. These mechanisms are related to oxygen radical overproduction during metabolic activation of tamoxifen [1]. Although the beneficial effects of tamoxifen are based on its effects as an estrogen antagonist in breast tissue, its use is associated with proestrogenic effects in other tissues, including increases in hepatic coagulation factor synthesis [2, 3]. Adverse outcomes noted with tamoxifen use include an increased incidence of cardiovascular events for example, deep vein thrombosis (DVT), pulmonary embolism, and stroke. Although the exact mechanism(s) for its prothrombotic effect is unknown, studies demonstrate that platelets treated with active metabolites of tamoxifen increase superoxide release through an NADPH oxidase-dependent mechanism and are associated with increased intracellular free calcium, leading to their activation [4, 5]. Contrary to this commonly noted observation, we report a case where, surprisingly, tamoxifen treatment was associated with decreased platelet activation and bleeding.

## 2. Case Report

We evaluated a 45-year-old female who presented with a 2-week history of easy bruising 4–6 weeks after taking only tamoxifen (20 mg/day). She was stage II (T1N1M0) left breast adenocarcinoma patient treated with partial mastectomy and axillary lymph node dissection followed by chemotherapy with adriamycin, cytoxan, and paclitaxel followed by irradiation. The patient's bruisability was associated with 45 min of bleeding after tooth extraction. She had no previous bleeding history that includes surgeries for a decompressive laminectomy and fusion, an iliac crest bone graft, bilateral breast reduction, ankle surgery, and the mastectomy. The patient denied oral ingestion of aspirin, nonsteroidal anti-inflammatory drugs (NSAIDs), or selective serotonin reuptake inhibitors (SSRIs), which are known to affect platelet function. Physical examination showed one large bluish-purple ecchymosis on the left leg and few smaller resolving bruises on both lower limbs. In the laboratory she had a normal complete blood count, renal and hepatic function, PT, APTT, fibrinogen, factor XIII, von Willebrand antigen and multimers, ristocetin cofactor assay, ristocetin-induced platelet aggregation, and no platelet-associated immunoglobulin. Platelet function studies revealed normal ADP-, epinephrine-, arachidonic acid-, and collagen-induced platelet aggregation and ATP release (Table 1). However, the patient had reduced mepacrine uptake but with normal release, suggesting an acquired storage pool disorder [6, 7]. It is recognized that patients with acquired storage pool disorders can have normal platelet aggregation and secretion studies [8, 9]. After tamoxifen was stopped, the patient's bruising ameliorated. Repeat platelet function studies performed 3 months later revealed normal platelet function studies and full correction of the mepacrine uptake defect (Table 1).

## 3. Discussion

These studies suggest the development of an acquired storage pool disorder with a reversible reduction in dense granule uptake associated with tamoxifen therapy. Platelet storage pool disorders arise from decreased platelet granule content of dense, alpha, or combined granules that are associated with reduced platelet function and bleeding. Dense granules contain 5-hydroxytryptamine (serotonin) and ATP/ADP [8, 9]. Mepacrine is a dense granule marker used for flow cytometry [10]. Quantitative reduction of any dense granule constituent is sufficient for the diagnosis of a storage pool disorder [8–10]. Although inherited platelet storage pool defects are rare, acquired platelet storage pool disorders are a more common occurrence seen in conjunction with autoimmune disorders such as systemic lupus erythematosus, cardiovascular bypass, and hematological disorders such as hairy cell leukemia, myelodysplasia, and myeloproliferative disorders [11–13]. In vivo platelet activation followed by reduction in circulating granule content release leads to a state of partial activation of platelets with acquired platelet dysfunction. Common entities associated with an acquired storage pool disorder include cardiac bypass with contact with the membrane oxygenator and immune complexes binding to platelets. Patients typically present with ‘‘platelet-type” hemorrhagic symptoms and signs such as mucosal membrane bleeding or prolonged bleeding postoperatively. Our report is the first to our knowledge to implicate an oral anticancer therapy producing an acquired platelet storage pool disorder.

Tamoxifen and Raloxifene, another selective estrogen receptor modulator (SERM), have been associated with increased platelet NO and reduced peroxynitrite leading to reduced platelet function [14, 15]. In experimental studies, tamoxifen treatment in micromolar concentrations inhibits all platelet functions with collagen activation being most sensitive, prolongs the bleeding time, and delays arterial thrombosis in vivo [16, 17]. Although the precise mechanism(s) contributing to an acquired storage pool disorder is not known, the ability of tamoxifen treatment to inhibit Ca^2+^ mobilization and IP3 phosphorylation suggest that there is reduced platelet activation leading to granule release [16, 17].

At the therapeutic dose of 20–40 mg/day, a concentration of 1 *μ*M of tamoxifen is achieved in the blood. Significant inhibition of platelet function, as noted by Nanetti et al., occurred only at tamoxifen levels of 5 *μ*M and above [14]. It is possible that our patient may be more sensitive to the antiplatelet effects of tamoxifen at therapeutic drug levels. Alternatively, the pharmacokinetics in this patient may have resulted in higher than usual levels of tamoxifen leading to the platelet dysfunction. Considering the effect of tamoxifen on platelet function, it is surprising that the observations noted in the present patient have not been previously appreciated.

## Figures and Tables

**Table 1:** Platelet aggregation and activation assays.

	12/08	3/09	Normal

Platelet aggregation			

ADP			
Percentage of aggregation	78	61	70–100% max
ATP release	0.85	0.23	0.2–1.6 nM
AA			
Percentage of aggregation	76	71	66–100% max
ATP release	0.68	0.29	0–1.3 nM
Collagen			
Percentage of aggregation	87	74	70–100% max
ATP release	1.02	0.53	0.4–1.9 nM
Epinephrine			
Percentage of aggregation	80	66	67–95% max
ATP release	1.02	0.54	0.3–1.7 nM

Platelet activation			

Percentage of mepacrine uptake	33	63	54–84%
Percentage of mepacrine release	82	79	58–93%

ADP: adenosine diphosphate; AA: arachidonic acid.
